# Chocolate and Health-Related Quality of Life: A Prospective Study

**DOI:** 10.1371/journal.pone.0123161

**Published:** 2015-04-22

**Authors:** Teresa Balboa-Castillo, Esther López-García, Luz M. León-Muñoz, Raúl F. Pérez-Tasigchana, José Ramón Banegas, Fernando Rodríguez-Artalejo, Pilar Guallar-Castillón

**Affiliations:** 1 Department of Public Health, School of Medicine, Universidad de la Frontera, Temuco, Chile; 2 Department of Preventive Medicine and Public Health, School of Medicine, Universidad Autónoma de Madrid/IdiPaz, CIBERESP, Madrid, Spain

## Abstract

**Background:**

Chocolate consumption has been associated with a short-term reduction in blood pressure and cholesterol, and improvement of insulin sensitivity; however, participants could not be aware of presenting hypertension or hypercholesterolemia. Moreover, the effect of chocolate on mental health is uncertain. This study assessed the association of regular chocolate consumption with the physical (PCS) and mental (MCS) components of health-related quality of life (HRQL).

**Materials and methods:**

We analyzed data from a cohort of 4599 individuals recruited in 2008–2010 and followed-up once prospectively to January 2013 (follow-up mean: 3.5 years). Regular chocolate consumption was assessed at baseline with a validated diet history. HRQL was assessed with the SF-12 v.2 at baseline and at follow-up. Analyses were performed with linear regression and adjusted for the main confounders, including HRQL at baseline.

**Results:**

At baseline, 72% of the study participants did not consume chocolate, 11% consumed ≤10 g/day and 17% >10 g/day. Chocolate consumption at baseline did not show an association with PCS and MCS of the SF-12 measured three years later. Compared to those who did not consume chocolate, the PCS scores were similar in those who consumed ≤10g/day (beta: -0.07; 95% confidence interval (95% CI): -0.94 to 0.80) and in those who consumed >10g/day (beta: 0.02; 95% CI:-0.71 to 0.75); corresponding figures for the MCS were 0.29; 95% CI: -0.67 to 1.26, and -0.57; 95%CI: -1.37 to 0.23. Similar results were found for sex, regardless of obesity, hypertension, hypercholesterolemia, diabetes or depression.

**Conclusions:**

No evidence was found of an association between chocolate intake and the physical or mental components of HRQL.

## Introduction

Chocolate is usually consumed in pleasant situations; many people find it delicious because chocolate has a characteristic texture, dissolves in the mouth, and has a nice aroma and a slightly bittersweet taste. From a nutritional standpoint, chocolate is energy-rich and has a high content of fat (saturated and to a lesser extent mono-unsaturated fat) and sugar. It also contains minerals (potassium, phosphorus, magnesium, and zinc), flavonols, biogenic amines (tyramine and phenylethylamine), methylxanthines (caffeine and theobromine), and cannabinoid-like fatty acids.[1]

Chocolate consumption, dark chocolate in particular, has been consistently associated with better physical health. Specifically, chocolate has been suggested to have short-term benefits on reducing blood pressure [2–4] and serum cholesterol, [4–7] and on improving insulin sensitivity; [4,8,9] also, in a number of long-term observational studies, chocolate consumption has been linked to lower incidence of cardiovascular disease.[2,10,11] However, participants do not usually perceive changes in biological risk factors for cardiovascular disease. Moreover, the effect of chocolate on mental health is uncertain because the few studies that have explored this issue have obtained conflicting results. [12–15]

Health-related quality of life (HRQL) represents the individual perception of well-being in several spheres of life, including physical and mental aspects. Poorer HRQL has been associated with greater use of healthcare services [16,17] and increased all-cause mortality. [18,19] To our knowledge, this is the first study to assess the prospective association of regular chocolate consumption with the physical and mental components of HRQL.

## Methods

### Study design and participants

Data were taken from a cohort of 6207 individuals aged 18 years and older. Cohort members were selected through random sampling of participants in the ENRICA study [20] with over-representation of older adults. Baseline data were collected in 2008–2010 in three stages: first, a phone interview with a structured questionnaire on health status, lifestyle, morbidity and health services use; second, a home visit to obtain biological samples (blood and urine); and third, another home visit to perform a physical exam and to conduct a diet history. Three years later (from May 2012 to January 2013; follow-up mean: 3.5 years), an attempt was made to contact the subjects again, which was successful in 4887 (78.7%) of cases. The socio-demographic, lifestyle and clinical characteristics were similar in subjects lost to follow-up and in those contacted. At follow-up, information was updated by phone interview using the same questionnaire as at baseline. All persons who collected information at baseline and at follow-up (nurses to obtain biological samples, and non-health personnel for the rest of the tasks) received specific training in the study procedures.

Study participants gave written informed consent. The study protocol was approved by the Clinical Research Ethics Committee of the *La Paz* University Hospital in Madrid.

### Chocolate consumption

Chocolate consumption during the year preceding the interview was collected with a validated computerized diet history developed from that used in the EPIC-Spain cohort study. [21,22] For chocolate, the energy-adjusted intraclass correlation coefficient between the diet history and the mean of seven 24-hour recalls was 0.49 in the validation study. Individuals reported the type of chocolate, the frequency and the amount consumed. Photographs were used to help estimate the size of the chocolate portion. Specifically, study participants were asked about the consumption of 25 foods containing chocolate; for this analysis, only milk chocolate bars, dark chocolate bars, unspecified chocolate, milk chocolate bars with nuts, thick hot chocolate, dark chocolate bars with nuts, bonbons, candy bars (e.g., Kit-Kat), white chocolate bars, candy bars (e.g., Mars, Snickers), chocolate for melting and truffles were considered. Finally, we calculated the total consumption of chocolate in g/day.

### Health related quality of life

HRQL was assessed with the Spanish version of the SF-12v.2, which has been validated. [23,24] The SF-12 is a shorter version of the SF-36 questionnaire, [25] and includes 12 items which allow for calculating a physical component summary (PCS) and a mental component summary (MCS), whose values are standardized to a national norm with a mean of 50 and a standard deviation of 10. Thus, the SF-12 summaries serve to compare the scores for each study participant against the mean score in the Spanish population. A higher score in the PCS or the MCS corresponds to better health status. [26]

### Potential confounders of the study association

Study participants reported their sex, age, level of education, and consumption of tobacco and alcohol. Weight, height and waist circumference were measured under standardized conditions. [27] Body mass index (BMI) was calculated as weight in kg divided by the square of height in m. Abdominal obesity was defined as a waist circumference >102 cm in men and >88 cm in women. Leisure-time physical activity (MET-h/week) was estimated with the questionnaire used in the EPIC-Spain cohort study. [28] Sedentary behavior was approximated by the time (hours/week) spent watching TV. As regards diet, the total energy intake was calculated by using standard food composition tables, and accordance with the Mediterranean diet was assessed with the Trichopoulou index (0–2, 3–5, and 6–8 points for low, medium and high accordance, respectively). [29] Finally, study subjects reported the following physician-diagnosed diseases at baseline and at the end of follow-up: coronary heart disease, stroke, cancer at any site, depression requiring treatment, hypertension, diabetes, and hypercholesterolemia.

### Statistical analysis

Of the 4887 participants who were contacted at the time of the interview, 4780 were alive. Of these, we excluded 214 individuals who lacked data on chocolate consumption or HRQL, and 74 with missing data on other variables. Thus, the analyses were conducted with 4599 individuals.

The association of chocolate consumption in 2008–2010 with the PCS and MCS of the SF-12 in 2013 was summarized with regression coefficients and their 95% confidence intervals (CI), obtained from linear regression models. Chocolate consumption was categorized into three groups: no consumption (reference), ≤10 g/day and >10 g/day. Of note is that 10 g is the standard portion for chocolate intake in Spain. [30] In interpreting the study results, we considered that only regression coefficients greater than 3 points were clinically relevant. [24,26] The linear models were adjusted for HRQL and the rest of the above-mentioned potential confounders measured at baseline (2008–2010), and for incident diseases during follow-up (from 2008–2010 to 2012–2013).

Analyses were conducted in the total study sample and each sex separately, because the distribution of HRQL varies with sex. The analyses were also stratified by general obesity, hypertension, diabetes, hypercholesterolemia, and depression to assess if these variables (which could be influenced by chocolate intake) mediate or modify the study association. Due to limited sample size, these analyses used chocolate consumption as a binary variable (yes vs. no).

Statistical significance was set at a 2-sided p <0.05. Statistical analyses were conducted with Stata v.11.

## Results

At baseline, 72% of the study participants did not consume chocolate, 11% consumed ≤10 g/day and 17% >10 g/day. Among those who consumed chocolate, mean intake was 23.2 g/day (24.7 in men and 22.2 in women). Milk chocolate and dark chocolate bars were the varieties with the highest consumption (Table 1)

**Table 1:** Chocolate consumption in g/day for the study participants in the ENRICA cohort study (2008–2010) by type of chocolate, among chocolate consumers.

	Total (n = 1272)		Men (n = 528)		Women (n = 744)	
Types of chocolate	Mean	SD	Mean	SD	Mean	SD
Milk chocolate bars	8.0	25.3	8.3	31.1	7.8	20.3
Dark chocolate bars	5.0	12.8	4.6	12.5	5.1	13.0
Unspecified chocolate	3.2	15.4	4.2	19.8	2.5	11.1
Milk chocolate bars with nuts	3.0	10.7	3.3	10.4	2.7	10.9
Thick hot chocolate	1.7	12.3	1.6	13.6	1.7	11.2
Dark chocolate bars with nuts	0.9	4.7	1.0	5.0	0.7	4.5
Bonbons	0.4	2.6	0.2	1.3	0.5	3.3
Candy bars (e.g., Kit-Kat)	0.4	4.3	0.6	6.1	0.1	2.4
White chocolate bars	0.3	2.7	0.1	1.6	0.4	3.3
Candy bars (e.g.,Mars, Snickers)	0.2	2.4	0.2	3.5	0.1	1.0
Chocolate for melting	0.1	1.9	0.1	2.3	0.1	1.7
Truffles	0.1	0.7	—	—	0.1	1.0
Total	23.2	22.1	24.7	38.2	22.2	27.0

SD: Standard Deviation

Compared to those with no chocolate consumption, consumers were more frequently women, younger, and had higher education; they also showed lower alcohol intake, BMI and frequency of abdominal obesity, but higher energy intake. As regards to baseline HRQL, the PCS and the MCS varied little across the categories of chocolate consumption when both crude and adjusted means were considered (Table 2).

**Table 2:** Baseline characteristics for the study participants in the ENRICA cohort study (2008–2010) according to categories of chocolate consumption.

			Chocolate consumption (g/d)			
		Overall	No consumption	≤ 10 g/day	> 10 g/day	p value
**Participants** (n)		4599	3327	479	793	
**Total chocolate consumption,** g/day, mean (SD)		6.4 (19.8)	0	5.1 (2.5)	34.1 (36.5)	<0.0001
**Men,** %		49.2	52.1	40.3	42.2	<0.0001
**Age,** mean (SD)		54.1 (17.2)	55.3 (17.9)	55.1(16.44)	51.1 (17.2)	<0.0001
**Educational level,** %						
No formal or primary education		36.7	39.6	32.8	27.1	<0.0001
Secondary education		36.4	34.8	38.0	41.9	
University		26.9	25.6	29.2	30.9	
**Tobacco consumption,** %						
Never smokers		49.3	23.6	20.0	23.6	0.01
Former smokers		27.4	28.3	23.6	26.0	
Current smokers		23.24	48.1	56.4	50.4	
**Alcohol consumption,** g/day, mean (SD)		12.3 (20.1)	13.2 (21.0)	9.6 (16.1)	10.6 (10.5)	<0.0001
**Body mass index,** Kg/m^2^, mean (SD)		27.4 (4.6)	27.6 (4.5)	27.0 (4.4)	26.9 (4.8)	<0.0001
**Abdominal obesity,** %		42.8	43.9	38.8	40.7	<0.0001
**Leisure time physical activity,** METs h/week, mean (SD)		26.4 (20.4)	26.3 (20.3)	27.1 (21.4)	26.2 (20.0)	0.67
**Time spent watching TV,** (h/week), mean (SD)		15.0 (10.2)	15.2 (10.2)	15.0 (10.5)	14.8 (9.9)	0.55
**Adherence to the Mediterranean diet,** * %						
	Low adherence (0–2 points)	15.7	16.3	11.7	22.6	<0,0001
	Medium adherence (3–5 points)	58.5	61.5	66.0	60.0	
	High adherence (6–8 points)	25.7	22.2	22.3	18.0	
**Total energy intake,** kcal/day, mean (SD)		2167 (624)	2101 (617)	2166 (525)	2446 (635)	<0.0001
**Prevalent diseases at baseline,** %						
Coronary heart disease		0.8	0.9	0.6	0.4	0.20
Stroke		0.6	0.6	0.6	0.5	0.94
Cancer		1.2	1.4	0.6	0.8	0.10
Depression		6.9	6.9	6.5	7.3	0.85
**Incident diseases during follow-up,** %						
Coronary heart disease		0.7	0.6	0.6	0.8	0.92
Stroke		0.8	0.9	0.2	0.8	0.30
Cancer		1.9	2.1	2.5	1.0	0.09
Depression		6.8	6.8	6.5	7.1	0.92
**Cardiovascular risk factors,** %						
	Hypertension **	44.0	45.8	39.6	39.1	0.0004
	Diabetes†	9.3	10.4	8.5	5.6	0.0002
	Hypercholesterolemia‡	59.1	59.6	58.3	56.7	0.30
**Physical component summary,** mean (SD)		48.9 (10.8)	48.7 (10.9)	49.2 (9.6)	49.7 (10.2)	0.37
**Mental component summary,** mean (SD)		51.1 (10.2)	51.1 (10.4)	51.8 (9.1)	50.6 (9.8)	0.57
**Physical component summary,** mean (SD)║		48.9 (9.4)	48.9 (9.5)	48.7 (9.5)	48.9 (9.7)	0.93
**Mental component summary,** mean (SD)║		51.0 (9.5)	50.9 (9.6)	51.8 (9.5)	50.9 (9.7)	0.56

SD: Standard Deviation; MET: Metabolic Equivalents; TV: television;

* Trichopoulou score.

** Hypertension was defined as systolic pressure ≥140 mm Hg, diastolic pressure ≥90mmHg, or use of antihypertensive drugs.

†Diabetes mellitus was defined as serum glucose ≥126 mg/dl or use of antidiabetic drugs.

‡Hypercholesterolemia was defined as total serum cholesterol ≥200 mg/dl or use of hypolipidemic drugs.

║Adjusted for sex, age (18–44,45–64,65 and over), educational level (no formal or primary education; secondary education; university), tobacco consumption (never smokers, former smokers, current smokers), alcohol consumption g/day (quartiles), body mass index kg/m^2^ (quartiles), abdominal obesity (yes/no), leisure time physical activity METs-h/week (quartiles), time spent watching television h/week (quartiles), adherence to the Mediterranean diet (low adherence 0–2 points, medium adherence 3–5 points, high adherence 6–8 points), total energy kcal/day (quartiles) and prevalent diseases (coronary heart disease, stroke, cancer and depression).

In the adjusted analyses, chocolate consumption at baseline did not show a statistically or clinically significant association with either the PCS or the MCS scores three years later. Compared to those who did not consume chocolate, the PCS scores were similar in those who consumed ≤10 g/day (beta: -0.07; 95% confidence interval (CI): -0.94 to 0.80) and in those who consumed >10 g/day (beta: 0.02; 95% CI: -0.71 to 0.75); corresponding figures for the MCS were 0.29; 95% CI: -0.67 to 1.26, and -0.57; 95% CI: -1.37 to 0.23. Similar results were found in each sex (Table 3).

**Table 3:** Linear regression coefficients (95% confidence interval) of the physical and mental components of the SF-12 in 2012–13 by chocolate consumption categories.

Chocolate consumption (g/day)	n	Physical component summary	Mental component summary
**Total participants**			
No consumption	3327	1.00 (Ref.)	1.00 (Ref.)
≤ 10 g/day	479	-0.07 (-0.94 to 0.80)	0.29 (-0.67 to 1.26)
> 10 g/day	793	0.02 (-0.71 to 0.75)	-0.57 (-1.37 to 0.23)
p for trend		0.87	0.32
**Men**			
No consumption	1734	1.00 (Ref.)	1.00 (Ref.)
≤ 10 g/day	193	-0.06 (-1.35 to 1.22)	-0.17 (-1.53 to 1.19)
> 10 g/day	335	0.001 (-1.04 to 1.04)	0.27 (-0.82 to 1.37)
p for trend		0.98	0.70
**Women**			
No consumption	1593	1.00 (Ref.)	1.00 (Ref.)
≤ 10 g/day	286	0.006 (-1.20 to 1.21)	0.95 (-0.43 to 2.32)
> 10 g/day	458	0.09 (-0.93 to 1.11)	-0.83 (-2.01 to 0.34)
p for trend		0.87	0.32

*p <0,05;

** p <0,01.

Model adjusted for sex, age (18–44,45–64,65 and over), educational level (no formal or primary education; secondary education; university), tobacco consumption (never smokers, former smokers, current smokers), alcohol consumption g/day (quartiles), body mass index kg/m^2^ (quartiles), abdominal obesity (yes/no), leisure time physical activity METs-h/week (quartiles), time spent watching television h/week (quartiles), adherence to the Mediterranean diet (low adherence 0–2 points, medium adherence 3–5 points, high adherence 6–8 points), total energy kcal/day (quartiles), prevalent diseases (coronary heart disease, stroke, cancer and depression), incident diseases (coronary heart disease, stroke, cancer and depression), physical or mental component summaries at baseline, as appropriate.

When chocolate consumption was classified as a dichotomous variable, the adjusted-mean of the PCS three years later was 48.1 among participants without chocolate consumption and 48.2 among those who consumed any amount of chocolate (p-value = 0.78). The corresponding figures for the MCS were: 51.5 and 51.4 (p-value = 0.76)."

Lastly, no association was observed between chocolate consumption and HRQL when the analyses were stratified by general obesity, hypertension, hypercholesterolemia, diabetes mellitus, and depression (Table 4).

**Table 4:** Linear regression coefficients (95% confidence interval) of the physical and mental components of the SF-12 in 2012–13 by chocolate consumption categories, stratified by educational level, lifestyles and prevalent diseases.

Stratified models		Chocolate consumption	n	Physical component summary	Mental component summary
**Educational level**					
	No formal or primary education	**No**	1318	1.00 (Ref.)	1.00 (Ref.)
		**Yes**	372	-0.58 (-1.79 to 0.63)	0.19 (-1.11 to 1.50)
	Secondary education or univesity	**No**	2009	1.00 (Ref.)	1.00 (Ref.)
		**Yes**	900	0.23 (-0.44 to 0.90)	-0.44 (-1.20 to 0.32)
**Leisure time physical activity**					
	Below the median	**No**	1681	1.00 (Ref.)	1.00 (Ref.)
		**Yes**	648	0.23 (-0.69 to 1.15)	-0.69 (-1.67 to 0.29)
	Above the median	**No**	1646	1.00 (Ref.)	1.00 (Ref.)
		**Yes**	624	-0.30 (-1.09 to 0.49)	0.32 (-0.59 to 1.23)
**General obesity**					
	With general obesity (BMI ≥30 kg/m^2^)	**No**	880	1.00 (Ref.)	1.00 (Ref.)
		**Yes**	266	-0.72 (-2.15 to 0.71)	-0.74 (-2.28 to 0.79)
	Without general obesity (BMI <30 kg/m^2^)	**No**	2447	1.00 (Ref.)	1.00 (Ref.)
		**Yes**	1006	0.22 (-0.44 to 0.87)	-0.03 (-0.76 to 0.71)
**Hypertension**					
	With hypertension	**No**	1514	1.00 (Ref.)	1.00 (Ref.)
		**Yes**	496	-0.10 (-1.13 to 0.93)	0.14 (-0.93 to 1.21)
	Without hypertension	**No**	1813	1.00 (Ref.)	1.00 (Ref.)
		**Yes**	776	0.09 (-0.64 to 0.82)	-0.42 (-1.28 to 0.44)
**Diabetes**					
	With diabetes	**No**	341	1.00 (Ref.)	1.00 (Ref.)
		**Yes**	84	1.24 (-1.43 to 3.91)	1.97 (-0.72 to 4.67)
	Without diabetes	**No**	2986	1.00 (Ref.)	1.00 (Ref.)
		**Yes**	1188	-0.12 (-0.73 to 0.50)	-0.36 (-1.04 to 0.33)
**Hypercholesterolemia**					
	With hypercholesterolemia	**No**	1980	1.00 (Ref.)	1.00 (Ref.)
		**Yes**	545	0.11 (-0.72 to 0.95)	-0.44 (-1.35 to 0.46)
	Without hypercholesterolemia	**No**	1347	1.00 (Ref.)	1.00 (Ref.)
		**Yes**	727	0.06 (-0.80 to 0.92)	-0.27 (-1.26 to 0.72)
**Depression**					
	With depression	**No**	231	1.00 (Ref.)	1.00 (Ref.)
		**Yes**	89	-0.79 (-3.87 to 2.28)	-3.07 (-6.68 to 0.54)
	Without depression	**No**	3096	1.00 (Ref.)	1.00 (Ref.)
		**Yes**	1183	0.07 (-0.54 to 0.68)	-0.05 (-0.72 to 0.62)

*p <0,05;

** p <0,01.

Model adjusted for sex, age (18–44,45–64,65 and over), educational level (no formal or primary education; secondary education; university), tobacco consumption (never smokers, former smokers, current smokers), alcohol consumption g/day (quartiles), body mass index kg/m^2^ (quartiles), abdominal obesity (yes/no), leisure time physical activity METs-h/week (quartiles), time spent watching television h/week (quartiles), adherence to the Mediterranean diet (low adherence 0–2 points, medium adherence 3–5 points, high adherence 6–8 points), total energy kcal/day (quartiles), prevalent diseases (coronary heart disease, stroke, cancer and depression), incident diseases (coronary heart disease, stroke, cancer and depression), physical or mental component summaries at baseline, as appropriate.

## Discussion

In this 3-year prospective study in the adult population of Spain, chocolate consumption did not show an association with either the physical or the mental components of HRQL.

As regards the potential effect of chocolate on physical health, chocolate consumption lowers blood pressure and cholesterol, and improves insulin sensitivity and vascular function. [2–9] However, it seems that the effects of chocolate on blood pressure are apparent mostly in individuals with hypertension or prehypertension, [31,32] and the improvement in lipid profile resulting from chocolate intake has been observed specifically in those with cardiovascular disease risks. [6] Of note, we found no association between chocolate and better physical HRQL, even in subjects diagnosed with hypertension, hypercholesterolemia or diabetes. Moreover, in a recent small trial with 37 g/day of dark chocolate administered during 6 weeks, no effect of chocolate was observed on blood pressure, serum lipids or C-reactive protein; chocolate consumers only showed higher pulse rates at 3 and at 6 weeks. [15] Lastly, the type of cocoa processing modifies the nutritional content of chocolate, particularly of the dark and white varieties, reducing the level of flavonoids and the antioxidant capacity. [33,34] In any case, these effects are not usually perceived, and the diagnosis of hypertension or hypercholesterolemia in frequent results from periodic screening or is a casual finding. Thus, the effect of chocolate on these biological factors may not translate into a better health perception. [35,36]

Although the negative effect of obesity on HRQL is well documented, [37] the association between chocolate intake and obesity is still uncertain. In fact, while chocolate is energy-rich and is frequently consumed as a snack, two cross-sectional studies have found an inverse association between chocolate intake and BMI. [38,39]

As regards chocolate and mental health, three cross-sectional studies have reported inconsistent associations. The first study was an exploratory investigation with 362 Swedish women, in which higher intake of sweet foods (specifically chocolate) correlated with more severe psychiatric symptomatology. [12] In the second study, which was conducted with over 1300 elderly men in Finland, those with a preference for chocolate over other types of candy reported better scores for subjective health, feelings of happiness and loneliness, and the Zung depression scale. [13] In contrast, a third study with 1018 adults from the U.S. showed that a higher consumption of chocolate was associated with a better score on the Center for Epidemiologic Studies Depression scale. [14]

Our results are in line with those from a trial with 101 subjects, which did not observe any effect of 37 g/day of chocolate during 6 weeks on any neuropsychological variables, including memory, thinking processes, mood and energy.[15] Several researchers have emphasized that the possible benefits of chocolate on mood, if any, are short-lived. [40–43] Match et al. reported that the mood effects of chocolate last for only 3 minutes, and derive mostly from its palatability. [43] Another study found that the reduced tiredness associated with chocolate intake lasts for only 1 hour. [44] In fact, phenylethylamine (a component of chocolate involved in the pathogenesis of depression) is metabolized quickly, with a half-life of only 5–10 minutes. [45] Therefore, Benton *et al*. concluded that the levels of psychoactive substances provided by chocolate were several orders of magnitude less than those needed to produce a pharmacological action. [46]

It has been suggested that depressed individuals may consume chocolate as a sort of "self-medication," which temporarily relieves symptoms by increasing serotonin centrally, due to the effect of tryptophan contained in chocolate. [47] Our results, however, showed no association between chocolate consumption and better health among those with diagnosed depression.

This study had some strengths and limitations. Among the strengths are the longitudinal design and the large sample of community-dwelling adults. Moreover, chocolate intake and HRQL were assessed with validated instruments. Lastly, the analyses accounted for a good number of potential confounders.

The main limitation was that diet was self-reported; therefore certain underreporting cannot be ruled out, particularly among the obese. Thus, obese participants could underestimate chocolate consumption because of a social desirability bias. Given that obesity is associated with poorer HRQL, this could lead the study association to the null. However, the main results were also found among the individuals with normal BMI. Another limitation is that chocolate could have been consumed for a longtime before the diet measurement at baseline. Our analyses also assume that chocolate consumption stays similar throughout the follow-up period. However, these limitations would be of greater importance had the observed association been positive, because it could be due to chocolate consumption before or after the study baseline. In any case, our results should be confirmed by an appropriately conducted clinical trial Finally, chocolate consumption in the study sample (and in Spain as a whole) was relatively low; the average consumption among chocolate consumers was 23.2 g/day, which is substantially lower than in central European countries and the U.S. [48] Thus, our results may not apply to higher chocolate consumers such as those in these countries.

In conclusion, in this large prospective study, we have found no evidence of beneficial effects of regular consumption of chocolate on HRQL. However, our results do not exclude a transient association between chocolate and certain dimensions of subjective health and wellbeing, which merit further investigation.
